# Supplementary material for: From sequence to enzyme mechanism using multi-label machine learning
Source: BMC Bioinformatics. 2014 May 19;15:150. doi: 10.1186/1471-2105-15-150 (PMC4229970; doi:10.1186/1471-2105-15-150)
Supplement: Additional file 2 — Java code of ml2db. Additional file ml2db_code.tar.gz contains the Java source code to run the multi-label machine learning experiments and save the results to database. The code’s Javadoc is included. [file 1471-2105-15-150-S2.zip › additional file 2/ml2db/ecmulan/doc/uk/ac/ed/inf/mulanxml/XmlCreator.html]

XmlCreator


JavaScript is disabled on your browser.


- Overview
- Package
- Class
- Use
- Tree
- Deprecated
- Index
- Help

- Prev Class
- Next Class

- Frames
- No Frames

- All Classes

- Summary:
- Nested |
- Field |
- Constr |
- Method

- Detail:
- Field |
- Constr |
- Method


uk.ac.ed.inf.mulanxml

## Class XmlCreator

- java.lang.Object
- - uk.ac.ed.inf.mulanxml.XmlCreator

- Direct Known Subclasses:
  :   EcFullXmlCreator

  ---

    

  ```
  public class XmlCreator
  extends java.lang.Object
  ```

  Class

  Version:
  :   19 Jun 2012

  Author:
  :   Luna De Ferrari luna.deferrari@ed.ac.uk or
      luna.deferrari@st-andrews.ac.uk

- - ### Constructor Summary

    Constructors

    Constructor and Description

    `XmlCreator(XmlCreatorManager manager,
    java.util.TreeSet<java.lang.String> labels)`

    Get labels from database
  - ### Method Summary

    Methods

    | Modifier and Type | Method and Description |
    | --- | --- |
    | `java.lang.String` | `createXmlString()` Generate a flat XML representation of the labels in Mulan format. |
    | `java.util.TreeSet<java.lang.String>` | `getLabels()` |
    | `XmlCreatorManager` | `getManager()` |
    | `MulanXml` | `getXml()` |
    | `java.lang.String` | `getXmlString()` Adds a log to the xml file (timestamp, database and query to the data etc.) |
    | `void` | `saveToFile(java.lang.String filePath)` |

    - ### Methods inherited from class java.lang.Object

      `equals, getClass, hashCode, notify, notifyAll, toString, wait, wait, wait`

- - ### Constructor Detail


    - #### XmlCreator

      ```
      public XmlCreator(XmlCreatorManager manager,
                java.util.TreeSet<java.lang.String> labels)
      ```

      Get labels from database

      Parameters:
      :   `dbConnPropsPath` -
  - ### Method Detail


    - #### getXml

      ```
      public MulanXml getXml()
      ```

      Returns:
      :   m\_xml


    - #### getLabels

      ```
      public java.util.TreeSet<java.lang.String> getLabels()
      ```

      Returns:
      :   m\_labels


    - #### getManager

      ```
      public XmlCreatorManager getManager()
      ```

      Returns:
      :   m\_manager


    - #### getXmlString

      ```
      public java.lang.String getXmlString()
      ```

      Adds a log to the xml file (timestamp, database and query to the data
      etc.)

      Returns:


    - #### createXmlString

      ```
      public java.lang.String createXmlString()
      ```

      Generate a flat XML representation of the labels in Mulan format.

      Returns:
      :   a flat XML representation of the labels in Mulan format.


    - #### saveToFile

      ```
      public void saveToFile(java.lang.String filePath)
      ```


- Overview
- Package
- Class
- Use
- Tree
- Deprecated
- Index
- Help

- Prev Class
- Next Class

- Frames
- No Frames

- All Classes

- Summary:
- Nested |
- Field |
- Constr |
- Method

- Detail:
- Field |
- Constr |
- Method
